# Intellectual development in Noonan syndrome: a longitudinal study

**DOI:** 10.1002/brb3.479

**Published:** 2016-05-03

**Authors:** Renée L. Roelofs, Nikki Janssen, Ellen Wingbermühle, Roy P.C. Kessels, Jos I.M. Egger

**Affiliations:** ^1^ Centre of Excellence for NeuropsychiatryVincent van Gogh Institute for PsychiatryVenrayThe Netherlands; ^2^ Centre of Excellence for Korsakoff and Alcohol‐Related Cognitive DisordersVincent van Gogh Institute for PsychiatryVenrayThe Netherlands; ^3^ Donders Institute for BrainCognition and BehaviorRadboud University NijmegenNijmegenThe Netherlands; ^4^ Behavioral Science InstituteRadboud University NijmegenNijmegenThe Netherlands; ^5^ School of Psychology and Artificial IntelligenceRadboud University NijmegenNijmegenThe Netherlands; ^6^ Department of Medical PsychologyRadboud University Medical CenterNijmegenThe Netherlands

**Keywords:** Cognition, contextual neuropsychology, intelligence, longitudinal design, neurodevelopmental disorder, Noonan syndrome, RAS‐MAPK

## Abstract

**Introduction:**

Although cognitive impairments in adults with Noonan syndrome seem to be limited to a low‐average intelligence and slower processing speed, studies in children with Noonan syndrome have demonstrated more extensive cognitive problems. These include deficits in language skills, memory, attention, and executive functioning. This longitudinal study is the first to investigate intellectual development in a group of individuals with Noonan syndrome.

**Methods:**

Sixteen patients with Noonan syndrome underwent intelligence assessment both in childhood and in adulthood, using Wechsler's intelligence scales. IQ scores and Wechsler standard scores achieved in childhood and adulthood were compared. Subsequently, verbal and performance IQ in childhood were used as predictors for adult IQ and index scores.

**Results:**

Compared with childhood scores, adult full‐scale IQ and performance IQ significantly increased. Adult performance IQ was higher than verbal IQ. Childhood performance IQ and verbal IQ together predicted all adult IQ and index scores, except for the processing speed index.

**Discussion:**

Childhood IQ was a significant predictor of adult intelligence in patients with Noonan syndrome. Performance IQ advanced to a normal level in adulthood, while verbal IQ did not develop proportionately, resulting in a discrepancy between adult performance IQ and verbal IQ. This finding could suggest a delay in the development of executive functioning in patients with Noonan syndrome, which seems to be outgrown in adulthood.

## Introduction

Noonan syndrome (NS) is a common autosomal dominant genetic disorder, caused by gene mutations in the Ras‐mitogen‐activated protein kinase (RAS‐MAPK) pathway, a signal transduction cascade involved in cell proliferation, differentiation, survival, and metabolism (Roberts et al. 2013). So far, mutations in fourteen genes (*PTPN11*,* SOS1*,* SOS2*,* RAF1*,* RIT1*,* KRAS*,* BRAF*,* NRAS*,* A2ML1*,* LZTR1*,* SHOC2*,* CBL*,* MAP2K1*,* RRAS*) have been associated with NS or Noonan‐like disorders (Aoki et al. 2013; Cordeddu et al. 2015; Flex et al. 2014; Pasmant et al. 2009; Vissers et al. 2015; Yamamoto et al., 2015). With regard to the phenotypic presentation, NS is characterized by short stature, distinct facial features, a webbed neck, and heart defects (Tartaglia et al. 2010). Although the main research topics in NS include genetic aspects and somatic complications, cognitive functioning has received increased scientific attention during the past decade.

Intelligence has been studied most frequently. In general, low‐average scores are found, with a wide range from intellectual disability to superior intelligence present in some patients. Overall, verbal and performance intellectual capacities seem to be evenly distributed (Wingbermühle et al. 2009). Despite only a low‐average mean intelligence, almost half of the children with NS have special educational needs (Van der Burgt et al. 1999; Shaw et al. 2007; Wingbermühle et al. 2012b).

In previous studies measuring cognitive performance in children with NS, impairments have been reported in one or more of the following domains: visual processing, memory, language functioning, communication, attention, motor functioning, and executive functioning (Wingbermühle et al. 2009; Pierpont et al. 2010, 2013, 2015; Alfieri et al. 2011a,b). It should be noted, however, that most studies in children with NS did not include controls matched on age, sex, and intelligence level. Therefore, the reported cognitive problems in children with NS could be related to their lower intellectual abilities rather than reflect Noonan‐specific cognitive impairments.

Regarding cognition in adults with NS, Wingbermühle et al. (2012b) only found a slower speed of information processing in comparison with controls matched with respect to age, sex, intelligence, and education level. Performance on objective measures of other cognitive domains such as memory, executive functioning, and visuoconstruction was relatively intact, although patients reported more cognitive complaints on self‐report questionnaires than controls.

In sum, children with NS seem to display more diffuse cognitive deficits as compared to adults with NS. Children with NS possibly outgrow their cognitive impairments, but to date, no longitudinal group studies have been performed to confirm this. Through a collaboration with the department of Human Genetics of Radboud University Medical Center (Nijmegen, The Netherlands), unique intelligence data was gathered from of a sample of patients with NS who were tested both in childhood and in adulthood. The aims of this study are twofold. First, intelligence of adults with NS is compared with their childhood scores. In normally developing children, intelligence is considered to be a relatively stable construct after age six, although there is evidence for significant individual variation and change during development (McCall et al. 1973; Sternberg et al. 2001). Since the NS literature suggests more cognitive problems in children with NS compared with adults, it is hypothesized that this discrepancy will be reflected in the IQ scores. Therefore, lower IQ scores are expected in childhood in comparison with adult scores. Considering the slower speed of information processing that has been found in adults with NS, larger differences are expected in verbal IQ in contrast to performance IQ. Second, it is investigated whether adult IQ and index scores can be predicted based on childhood intelligence. As intelligence at age six is considered to be highly correlated with adult IQ (Bayley 1949), it is assumed that childhood intelligence in patients with NS will be predictive of adult IQ, despite the anticipated discrepancy between intellectual functioning in childhood and adulthood.

## Materials and Methods

### Participants

Inclusion criteria for participants comprised a confirmed clinical diagnosis of NS (Van der Burgt et al. 1994) and the availability of intelligence assessment both in childhood and in adulthood. In adulthood (>16 years), approximately fifty patients with NS underwent neuropsychological assessment at the Centre of Excellence for Neuropsychiatry of the Vincent van Gogh Institute for Psychiatry in Venray between 2006 and 2012. This assessment was part of the study of Wingbermühle et al. (2012a,b). If present, childhood neuropsychological data of these patients were retrieved. For a total of 16 patients, both childhood as well as adult intelligence data were available. Fourteen of these patients participated as children in the study of Van der Burgt et al. (1999), for which neuropsychological assessment was administered at the department of Pediatrics of Radboud University Medical Center, Nijmegen, between 1995 and 1997. Two participants underwent intelligence testing at other locations in 2002.

Out of the 16 included patients with NS (seven males/nine females), eight individuals had a confirmed mutation in *PTPN11* gene (50%), one in *SOS1*, and one in *MAP2K1*. In four patients with NS, no known mutation was found and in two patients no mutation analyses had been performed or completed yet. Mean age at assessment in childhood was 10.48 years (SD = 3.26, range = 6–16) and the average age at adulthood examination was 20.63 years (SD = 3.63, range = 16–27). The mean difference between the age at childhood assessment and adult examination was 10.15 years (SD = 2.15, 5–15). Twelve patients (75%) attended special education and 13 (81.25%) needed some kind of social support (e.g., assisted living facilities, social services). The level of education in adulthood was coded using seven categories according to the Dutch educational system, ranging from category 1 (1–5 years of education) to 7 (19–20 years of education) (Bouma et al. 2012). The level of education of adults with NS in the current sample ranged from 2 (only primary school completed) to 6 (5 years of high‐level secondary education).

Participation was voluntary and all participants and/or their legal representatives provided written informed consent prior to participation. In accordance with the Declaration of Helsinki, the study was approved by the Institutional Review Board of Vincent van Gogh Institute for Psychiatry.

### Materials and procedure

Full‐scale IQ, verbal IQ, and performance IQ in childhood (cFSIQ, cVIQ, cPIQ) were assessed by the Dutch Version of the Wechsler Intelligence Scale for Children Revised (WISC‐R: Van Haassen et al. 1986). The WISC‐R consists of six verbal subtests (*Information*,* Similarities*,* Arithmetic*,* Vocabulary*,* Comprehension*, and *Digit Span*) and six performance subtests (*Picture Completion*,* Picture Arrangement*,* Block Design*,* Object Assembly*,* Coding*, and *Mazes*) (Van Haassen et al. 1986). In 14 children, besides IQ scores, also Wechsler subtest scores were available.

In adulthood, IQ scores (aFSIQ, aVIQ, aPIQ) and index scores (Verbal Comprehension, Working Memory, Perceptual Organization, Processing Speed) of the Dutch version of the Wechsler Adult Intelligence Scale Third Edition were administered (WAIS‐III: Wechsler 2005a). aVIQ is composed of six subtests (*Vocabulary*,* Similarities*,* Arithmetic*,* Digit Span*,* Information*, and *Comprehension*) and measures acquired knowledge, verbal reasoning, and attention to verbal material. aPIQ is constructed from five subtests (*Picture Completion*,* Digit Symbol*,* Block Design*,* Matrix Reasoning*, and *Picture Arrangement*) and is considered to be a measure of fluid reasoning, spatial processing, attention to detail, and visuomotor integration. aFSIQ is the sum of aVIQ and aPIQ and provides an estimation of the general level of intellectual functioning (Wechsler 2005a). The Verbal Comprehension Index (aVCI) consists of three subtests and measures acquired verbal knowledge and reasoning. The aVCI is considered to be a more pure measure of verbal comprehension than aVIQ. The Perceptual Organization Index (aPOI) is composed of three subtests and measures fluid reasoning, attention to detail, and eye‐hand coordination. The aPOI is less sensitive for a slower processing speed than aPIQ, and is more focused on fluid reasoning and visual‐spatial problem solving skills. The Working Memory Index (aWMI) is constructed from three subtests and measures the ability to briefly memorize and process verbal information in order to repeat it. The Processing Speed Index (aPSI) is composed of two subtests and measures the speed by which visual information is being processed (Wechsler 2005a).

Although the WISC‐R and WAIS‐III are both validated instruments, highly comparable and structured, there are differences between the two tests (e.g., number of subtests). In order to assess the agreement between the WISC‐R and WAIS‐III, the literature was searched for correlation studies. In addition, Pearson, the publisher of the WISC‐R and WAIS‐III, has been consulted, but unfortunately no correlational data were available. Instead, the technical manual of the WAIS‐III only described the correlations between the WAIS‐III and WISC‐III (successor of WISC‐R). To get an impression, correlations between these IQ scores are high and range between 0.78 and 0.88. At subtest level, the correlations are moderate to high and range between 0.31 (Picture Arrangement) and 0.83 (Vocabulary) (Psychological Corporation, 1997).

### Statistical analysis

All statistical procedures were performed using SPSS version 22.0 (IBM Corp., Armonk, NY). Paired‐sample *t*‐tests were used to compare childhood and adult performance on the Wechsler intelligence scales. Seven multiple linear regression analyses were performed to predict adult IQ and index scores, based on cVIQ and cPIQ. cVIQ and cPIQ were included as predictors in the analyses and aFSIQ, aVIQ, aPIQ, aVCI, aWMI, aPOI, and aPSI were included as outcome variables. Preliminary analyses were conducted before performing the multiple regression analyses to ensure that the assumptions of normality, multicollinearity, and singularity were not violated. According to the Kolmogorov–Smirnov test, all variables were normally distributed.

## Results

### Comparison of IQ

After a qualitative comparison of the IQ scores in childhood and adulthood, a higher aFSIQ was found in 11 patients (68.75%), a lower aFSIQ in four patients, and the performance of one patient remained the same. The mean difference between childhood and adult scores was 5.13 (SD = 7.11). For aVIQ, the score of seven patients improved (46.75%), while it diminished in nine patients. The difference between the scores was on average −1.00 (SD = 8.15). Regarding aPIQ, 14 patients (87.5%) showed improved scores and only two patients achieved a lower score. The mean difference was 11.25 (SD = 8.20).

Childhood intelligence and the Wechsler subtest standard scores were statistically compared with adult performance by means of paired‐sample *t*‐tests (see Table 1). After Bonferroni correction (*α* = 0.02), FSIQ and PIQ appeared to be significantly higher in adulthood than in childhood (respectively, *t*(15) = 2.88, *P = *0.01 *η* ^2^ = 0.36; *t*(15) = 5.49, *P *<* *0.001, *η* ^2^ = 0.67). The difference in VIQ was not statistically significant (*t*(15) = −0.49, *P = *0.63).

**Table 1:** Mean, range, and standard deviation of Wechsler IQ and standard scores of children and adults with Noonan syndrome

	Children (WISC‐R)			Adults (WAIS‐III)		
	Mean	Range	SD	Mean	Range	SD
Full‐scale IQ^1^	83.50*	57–110	14.55	88.63	68–115	13.17
Verbal IQ^1^	87.88	59–110	14.39	86.88	65–113	12.55
Performance IQ^1^	81.75***	56–108	16.01	93.00	68–114	16.33
Picture completion^2^	9.36	5–14	2.76	10.00	4–15	3.40
Vocabulary^2^	8.29**	5–11	2.02	7.21	3–11	2.55
Digit symbol^2^	8.29	1–19	4.68	7.64	1–15	3.71
Similarities^2^	8.71	4–15	3.52	8.00	5–13	1.88
Block design^2^	7.07	2–11	2.62	8.43	4–13	3.06
Arithmetic^2^	7.71	2–13	3.43	8.43	4–13	2.74
Digit Span^2^	8.36	3–11	2.53	8.71	4–14	2.84
Information^2^	8.36	4–11	2.56	8.29	5–14	2.40
Picture arrangement^2^	7.93	3–13	2.89	10.07	3–15	3.79
Comprehension^2^	8.93	5–12	2.46	7.36	2–13	3.05
Mazes^2^	7.00	3–14	3.28	–	–	–
Object assembly^2^	7.14	1–14	3.76	–	–	–
Matrix reasoning^2^	–	–	–	9.50	4–15	3.48

WISC‐R, Wechsler Intelligence Scale for Children Revised; WAIS‐III, Wechsler Adult Intelligence Scale Third Edition; SD, standard deviation.

All *P*‐values are Bonferroni corrected, *P < 0.05, **P < 0.01, ***P < 0.001.

^1^Wechsler intelligence scores were available for 16 patients.

^2^Wechsler subtest standard scores were available for 14 patients.

At subtest level (adjusted *α* = 0.005), it appeared that only the performance on *Vocabulary* was significantly lower in adulthood than in childhood (*t*(13) = 3.74, *P *=* *0.002, *η* ^2^ = 0.48). The difference on the subtest *Comprehension* almost reached significance (*t*(13) = 3.22, *P = *0.007, *η* ^2^ = 0.41). There were no differences between childhood and adult performance on the other subtests.

**Figure 1:**
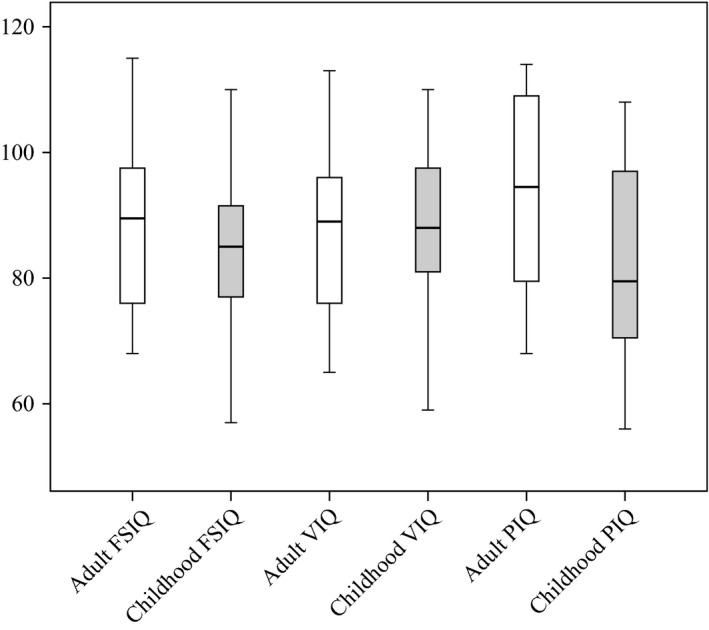
Boxplot of full scale, verbal, and performance IQ scores for Wechsler Intelligence Scale for Children Revised (WISC‐R) and Wechsler Adult Intelligence Scale Third Edition (WAIS‐III).

Within the childhood scores, the difference between VIQ and PIQ did not reach significance (*t*(15) = −1.75, *P *=* *0.10). However, within the adult scores, PIQ was significantly higher than VIQ (*t*(15) = −2.23, *P *=* *0.04, *η* ^2^ = 0.25). For a graphical display of the results, see Figure 1.

### Prediction of IQ scores

When cVIQ and cPIQ were simultaneously included in the regression analysis, the predictors combined had significant predictive power, explaining 79% of the variance in aFSIQ (*F*(2, 13) = 28.95, *P *<* *0.001). However, only cPIQ made a significant contribution to the prediction (*β* = 0.77, *P *<* *0.001), while the predictive value of cVIQ did not reach significance (*β* = 0.21, *P *=* *0.17). For all test statistics, see Table 2.

**Table 2:** Summary of the test statistics of the multiple regression analyses for adult Wechsler IQ and index scores (*N* = 16)

Predictors	*B*	SE (B)	95% CI for B	*β*	*t*	Sig. (*P*)
Full‐scale IQ (adj. *R* ^2^ = 0.79, *P* < 0.001)						
cVIQ	0.19	0.13	−0.10 to 0.48	0.21	1.44	0.17
cPIQ	0.63	0.12	0.37 to 0.89	0.77	5.23	<0.001
Verbal IQ (adj. *R* ^2^ = 0.79, *P* < 0.001)						
cVIQ	0.49	0.13	0.22 to 0.77	0.56	3.85	0.002
cPIQ	0.35	0.12	0.11 to 0.60	0.45	3.07	0.01
Performance IQ (adj. *R* ^2^ = 0.74, *P* < 0.001)						
cVIQ	−0.19	0.18	−0.58 to 0.21	−0.17	−1.02	0.33
cPIQ	0.99	0.16	0.63 to 1.34	0.97	6.01	<0.001
Verbal comprehension (adj. *R* ^2^ = 0.62, *P* = 0.001)						
cVIQ	0.49	0.16	0.16 to 0.83	0.62	3.16	0.01
cPIQ	0.21	0.14	−0.10 to 0.51	0.29	1.48	0.16
Working memory (adj. *R* ^2^ = 0.70, *P* < 0.001)						
cVIQ	0.38	0.17	0.01 to 0.75	0.38	2.20	0.05
cPIQ	0.52	0.16	0.18 to 0.85	0.58	3.34	0.01
Perceptual organization (adj. R^2^ = 0.68, *P* < 0.001)						
cVIQ	0.03	0.23	−0.47 to 0.52	0.02	0.11	0.91
cPIQ	0.95	0.21	0.51 to 1.39	0.84	4.62	<0.001
Processing speed (adj. *R* ^2^ = 0.24, *P* = 0.07)						
cVIQ	−0.35	0.35	−1.09 to 0.40	−0.28	−1.00	0.34
cPIQ	0.79	0.31	0.12 to 1.46	0.70	2.54	0.03

cVIQ, childhood Verbal IQ; cPIQ, childhood Performance IQ.

cVIQ and cPIQ together significantly predicted aVIQ, explaining 79% of the variance (*F*(2, 13) = 28.74, *P *<* *0.001). Both cVIQ and cPIQ made significant contributions to the prediction (respectively, *β* = 0.56, *P = *0.002; *β* = 0.45, *P = *0.009). Subsequently, two separate regression analyses were performed on the index scores underlying aVIQ: aVCI and aWMI. cVIQ and cPIQ together significantly predicted both index scores, explaining, respectively, 62% and 70% of the variance (aVCI: *F*(2, 13) = 13.32, *P = *0.001; aWMI: *F*(2, 13) = 18.62, *P < *0.001). cVIQ made a significant contribution to the prediction of aVCI (*β* = 0.62, *P = *0.008), while cPIQ was not significant (*β* = 0.29, *P = *0.16). Regarding aWMI, both cPIQ and cVIQ made significant contributions to the prediction (respectively, *β* = 0.58, *P = *0.005; *β* = 0.38, *P = *0.05).

The combination of cVIQ and cPIQ significantly predicted aPIQ as well, explaining 74% of the variance (*F*(2, 13) = 22.71, *P *<* *0.001). cPIQ made a significant contribution (*β* = 0.97, *P < *0.001), but cVIQ did not (*β* = −0.17, *P = *0.33). Next, the performance index scores aPOI and aPSI were included in two regression analyses. aPOI could significantly be predicted by cVIQ and cPIQ together, explaining 68% of the variance (*F*(2, 13) = 16.61, *P *<* *0.001). cPIQ appeared to have a strong significant contribution to the prediction (*β* = 0.84, *P < *0.001), but the contribution of cVIQ was not significant (*β* = 0.02, *P = *0.91). However, cVIQ and cPIQ together only showed a trend toward significance for predicting the aPSI (adjusted *R* ^2^ = 0.24, *F*(2, 13) = 3.40, *P *=* *0.07).

## Discussion

In this study, intellectual development was investigated in a group of patients with NS by comparing childhood IQ with adult intelligence levels. FSIQ and PIQ were significantly higher in adulthood compared with childhood scores, and a significant discrepancy was found between aVIQ and aPIQ, to the advantage of aPIQ. Furthermore, cVIQ and cPIQ together significantly predicted all adult IQ and index scores, except for aPSI.

Partly in accordance with the hypothesis, FSIQ and PIQ were significantly higher in adulthood in comparison with childhood scores. The FSIQ change was five IQ points on average, which is considered to be a small difference in clinical terms and would probably not result in a different classification. While PIQ appeared to increase to a normal level, VIQ did not develop proportionately, resulting in a VIQ‐PIQ discrepancy in adulthood. PIQ improved on average 11 IQ points, which is almost one standard deviation. VIQ on average decreased one IQ point. Although changes in FSIQ are often found in longitudinal studies of normally developing individuals, this specific increase in PIQ is remarkable and not in accordance with our hypothesis. Since Wingbermühle et al. (2012b) found a lower speed of information processing in adults with NS, only a modest improvement in PIQ was anticipated. Effects of repeated administration are mentioned as an explanation for improvements in PIQ in longitudinal studies (WISC‐III: Siders et al. 2006; WISC‐R: Tuma and Appelbaum 1980). However, the retest interval in this study is significantly larger than the maximum interval of six months that was used in these studies. Moreover, since different tests for childhood and adult testing were used, practice effects are considered to be highly unlikely.

The specific improvement in PIQ that was found in this sample may reflect a developmental delay in executive functioning in NS. PIQ is considered to be a measure of fluid reasoning, reflecting nonverbal problem solving skills in novel situations (Wechsler 2005a), which strongly overlaps with the construct of executive functioning. Executive functioning includes various cognitive processes, necessary for planning, organizing, and monitoring goal‐directed behavior (Decker et al. 2007; Van Aken et al. 2014), and is known to evolve gradually into adulthood. Moreover, childhood performance on the subtest Mazes, which is considered to represent planning capacities in children, was the lowest compared with the other subtests. An alternative explanation for finding a higher PIQ in adulthood concerns the maturation of motor functioning in NS. Mild motor delay has been frequently described in children with NS. These motor skills are thought to improve during development and could therefore provide an explanation for the increase in PIQ as well.

While PIQ improved in adulthood, VIQ did not develop proportionately. At subtest level, a significantly lower score on the subtest *Vocabulary* was found in adulthood. Verbal skills of children with NS are known to develop slower and many children with NS have articulation problems, for which they often receive speech therapy (Pierpont et al. 2010). It is often assumed that the process of getting more skilled in the use of language and speech through intensive repetition (naturally or by means of speech therapy) would resolve these difficulties in adulthood. However, the results of this study show that verbal reasoning remains weak in adulthood. There are indications that language and social cognition are interrelated domains and that language plays an important role in acquiring notion of abstract constructs such as emotions (Lindquist et al. 2015). Fujiki et al. (2002) also suggested that children with language impairments lack differentiation in the verbal expression of emotions and tend to simplify their internal feelings into broad terms. This is in accordance with a study of Lamberty and Holt (1995), in which a relation is described between deficits in developmental verbal skills and alexithymia. Future studies should investigate the predictive power of childhood verbal capacities on the development of alexithymic problems, which have been previously described in adults with NS.

The results of the regression analyses are in accordance with the general assumption that childhood intelligence is predictive of adult IQ. It was found that the combination of cVIQ and cPIQ could significantly predict aFSIQ, but only cPIQ made a significant contribution to the prediction. This finding could suggest that for patients with NS, (the development of) cPIQ, reflecting nonverbal reasoning abilities, may be a better indication of overall intellectual functioning later in life than acquired verbal knowledge. However, there could be a power problem due to the relatively small sample size in this study. Furthermore, the combination of cVIQ and cPIQ could also significantly predict aVIQ, aPIQ, aVCI, aWMI, and aPOI. Only aPSI could not be predicted by the combination of the two predictors, which could again reflect a lack of statistical power. Overall, it appeared that the predictor cPIQ had larger contributions than cVIQ to the prediction of aPIQ, aPOI, and aWMI, while cVIQ had larger contributions than cPIQ to the prediction of aVIQ and aVCI.

A significant strength of this study is its longitudinal design, since no other longitudinal group studies concerning intellectual or neuropsychological functioning have yet been performed in NS. In addition, the error variance associated with individual differences is reduced, because of the use of a within‐subject design.

Nonetheless, the results should also be interpreted in light of some limitations. First, even though a within‐subject study requires less participants than a between‐subject design to achieve good statistical power, the sample size of this study may still be considered relatively small, especially considering the heterogeneity that has been found in the neuropsychological phenotype of patients with NS. While a larger number of participants would allow more powerful statistical analyses, it should be noted that neuropsychological studies in NS mostly rely on convenience samples, and despite the fairly small sample, significant and relevant results could be reported here. Second, the presence of a selection bias cannot be ruled out, possibly affecting the generalization of these findings to the overall NS population. Children and adults with cognitive problems may have been more inclined to participate in these studies than those without subjective problems, which could have led to an over representation of individuals with cognitive complaints. Third, given the nature of this retrospective longitudinal study, no control data was available for the childhood IQ scores. Although the inclusion of a matched control group would be informative in terms of normal development, this study design still provides unique information concerning intellectual development in this specific population. Moreover, it is notable that this intelligence study in patients with NS again demonstrated a discrepancy between childhood and adult performance, which has been described in studies of cognitive functioning as well. This strengthens the belief that the current findings could reflect a typical NS‐related cognitive developmental delay that is outgrown in adulthood. However, controlled longitudinal studies should be performed to confirm this. Fourth, the non‐overlapping subtests of the WISC‐R and WAIS‐III could lead to subtle differences between the two tests in the extent to which they appeal to motor and executive functioning. Fifth, it is noted that both age groups contain an individual who was 16 years of age during the assessment. This overlap does, however, not impede our findings, as the age difference between childhood and adult assessment was at least five years for each individual, with a mean age difference of a little more than 10 years. Therefore, the presented predictive values are applicable for a period of at least five years.

In conclusion, the results of this study showed that childhood intelligence is a significant predictor of adult intelligence in individuals with NS and that PIQ develops more strongly than VIQ. This could suggest a developmental delay in underlying executive functioning in NS. Possibly, future neuroimaging studies may be of help to substantiate this neuropsychological hypothesis. Because VIQ tends to lag behind and does not develop proportionately with PIQ/executive functioning, monitoring and stimulating verbal skills in children with NS is recommended. As soon as the language skills in children with NS improved satisfactorily, educational attention should still go out to verbal reasoning abilities, because a deficit in this domain seems to persist in adulthood and may also hamper the verbalization of emotions. Although evaluation of cognitive functioning is implied in the clinical guidelines for the management of NS (Dyscerne, Noonan Syndrome Guideline Development Group, 2010), the present results highlight the importance of periodic re‐evaluation of cognitive functioning in NS.

## Conflict of Interest

None declared.
